# Quantitative Proteomic Analysis Reveals the Deregulation of Nicotinamide Adenine Dinucleotide Metabolism and CD38 in Inflammatory Bowel Disease

**DOI:** 10.1155/2019/3950628

**Published:** 2019-04-23

**Authors:** LongGui Ning, Guodong Shan, Zeyu Sun, Fenming Zhang, Chengfu Xu, Xinhe Lou, Sha Li, Haojie Du, Hongtan Chen, Guoqiang Xu

**Affiliations:** ^1^Department of Gastroenterology, First Affiliated Hospital, Zhejiang University School of Medicine, Hangzhou, China; ^2^Proteomics & Metabolomics Platform, State Key Laboratory for Diagnosis and Treatment of Infectious Diseases, The First Affiliated Hospital, Hangzhou, China

## Abstract

Inflammatory bowel disease (IBD) has become a major health challenge worldwide. However, the precise etiological and pathophysiological factors involved in IBD remain unclear. Proteomics can be used for large-scale protein identification analysis. In the current study, using tandem mass tag- (TMT-) based shotgun proteomics, proteomic differences between intestinal tissue from health controls, patients with Crohn's disease (CD), and patients with ulcerative colitis (UC) were compared. Proteins with fold change >2 or <0.5 and P value < 0.05 between groups were considered differentially expressed. ProteinAtlas was used to analyze the tissue specificity of differentially expressed proteins (DEPs). Reactome pathway analysis was applied to cluster functional pathways. A total of 4786 proteins were identified, with 59 proteins showing higher levels and 43 showing lower levels in patients with IBD than in controls. Seventeen proteins, including angiotensin converting enzyme 2 (ACE2) and angiotensin converting enzyme 1 (ACE), showed higher levels in CD than in UC. Several novel proteins such as CD38, chitinase 3-like 1 (CHI3L1), olfactomedin 4 (OLFM4), and intelectin 1 were screened out between patients with IBD and controls. When proteins with fold change >1.2 or <0.84 and P value < 0.05 between groups were considered differentially expressed, the expression of 10 proteins, including CD38, involved in the nicotinamide adenine dinucleotide (NAD) metabolism and signaling pathway showed significant changes in IBD. Using the NCBI GEO database, we confirmed increased CD38 mRNA expression in patients with UC and in mouse colitis models. Protein CD38 expression was higher in CD and UC than in normal controls. CD38 expression was higher in inflamed tissues than in noninflamed tissues, and CD38 was located in F4/80-positive cells. Our study may provide novel insights into the molecular pathogenesis of IBD. Further studies are required on the role of NAD metabolism and CD38 in intestinal inflammation.

## 1. Introduction

Inflammatory bowel disease (IBD) is categorized into Crohn's disease (CD) and ulcerative colitis (UC), which are characterized by relapsing chronic colitis in the gastrointestinal tract. An estimated 2.5 million people are affected by IBD in Europe [1]. In Asia, although the prevalence of IBD is lower than that in Europe, it has rapidly increased over the last decade [2, 3]. Thus, IBD has become a major health challenge worldwide. However, the precise etiological factors of IBD remain unclear. Currently, IBD is thought to result from interplay between environmental factors and host genetics, leading to persistent gastrointestinal immune activation [4, 5].

Various inflammatory molecules, including cytokines, chemokines, and danger-associated molecular patterns (DAMPs), are released from infiltrating inflammatory cells [4], and drugs targeting these inflammatory molecules are developed as therapeutics for IBD treatment [6]. Tumor necrosis factor-*α* (TNF-*α*) inhibitors are now the most commonly prescribed biologic therapeutics for patients with IBD. Other new therapeutic concepts such as Janus kinase (JAK) inhibitors, antiadhesion molecules, and anti-Smad7 have shown promising results in current clinical trials [7–9]. Much of the recent research in IBD has been focused on identifying novel molecules that may be therapeutic targets.

Currently, IBD diagnosis depends on clinical, endoscopic, radiographic, and laboratory findings. The differential diagnosis of CD and UC is clear in most cases; however, it is difficult to determine in an estimated 15% of patients because of atypical findings [10, 11]. Accurate diagnosis of IBD and differential diagnosis between UC and CD are essential for ensuring appropriate therapeutic intervention and surveillance [12]. Serological markers, especially perinuclear antineutrophil cytoplasmic antibodies (pANCAs) and anti–*Saccharomyces cerevisiae* antibodies (ASCAs), aid in differentiating UC from CD [13]; however, the sensitivity of this test is relatively low [14]. Histological biomarkers for this differential diagnosis are not well understood. Identifying molecules differentially expressed between CD and UC may help uncover the differences in their pathogenesis.

Proteomics helps provide novel strategies for large-scale protein identification analysis and valuable insights into disease pathophysiology. In the past decade, proteomic inquiries have helped uncover numerous host proteins and pathways related to IBD pathogenesis. Utilizing matrix-assisted laser desorption/ionization (MALDI)–time-of-flight (TOF) mass spectrometry (MS), Anna et al. [15] identified annexin A2 and programmed cell death protein 8 as being involved in the destruction of intestinal epithelial cell (IEC) homeostasis in UC. Zhao et al. [16] identified the p38 mitogen-activated protein kinase (MAPK) pathway as a molecular signature in UC. Moreover, serum proteomic panels have been used to differentiate CD from UC [17], to predict disease activity [18], and to evaluate response to infliximab (IFX) therapy [19]. In the current study, we aimed to identify potential proteins involved in IBD pathophysiology and to compare the proteomic differences between CD and UC by using tandem mass tag- (TMT-) based quantitative proteomics in order to identify novel proteins that may be associated with the pathogenesis of IBD and differentiation between CD and UC.

## 2. Materials and Methods

### 2.1. Sample Collection

The diagnostic criteria for both UC and CD were based on clinical, endoscopic, and histological features according to the World Gastroenterology Organization Practice Guidelines for IBD diagnosis and management [20]. For proteomic analysis, patients with CD (n = 9) or UC (n = 9) were recruited from inpatients of the Department of Gastroenterology, the First Affiliated Hospital of Zhejiang University. During colonoscopy, two intestinal tissue biopsy samples were obtained from the inflamed area. The normal controls were patients who underwent screening colonoscopies without active gastrointestinal pathology. Age and sex-matched normal control patients (n = 6) were recruited, and samples were obtained from the normal colon tissue during screening colonoscopy.

The independent groups established for validation were as follows: surgically resected colon tissues from three control patients, three patients with CD, and three patients with UC. For patients with CD or UC, both inflamed and noninflamed tissue samples were obtained. Information regarding baseline clinical characteristics was obtained during admission. Informed consent was obtained from all subjects before participation. The study protocol conforms to the ethical guidelines of the 1975 Declaration of Helsinki (6th revision, 2008), as reflected in the approval by the ethics committee of the First Affiliated Hospital of Zhejiang University School of Medicine. In all cases, colonoscopy biopsy or resected colon tissue specimens were rinsed in phosphate-buffered saline and immediately frozen in liquid nitrogen before storage at -80°C.

### 2.2. Protein Extraction and Digestion

Tissues were homogenized in radioimmunoprecipitation assay (RIPA) lysis buffer and were centrifuged. Protein concentrations were measured with the bicinchoninic acid (BCA) assay (Beyotime, Beijing, China). All samples were reduced with dithiothreitol (10 mM) at 60°C for 30 min and alkylated with iodoacetamide (30 mM) for 30 min at room temperature in the dark. The proteins were then incubated with cold acetone for 4 h at 0°C. The protein pellets were centrifuged at 3000 rpm for 15 min at 4°C and resuspended in 50 *μ*M triethylammonium bicarbonate (TEAB). Trypsin (Thermo Fisher Scientific, America) was added at a 1:50 enzyme: substrate ratio for overnight digestion at 37°C. For TMT labeling, 24 samples, each containing 30 *μ*g protein digest, were divided into three TMT experiments. A common reference sample, created by equal mixing of all samples, was labeled with TMT-131 and TMT-130C across all 3 TMT experiments. The TMT-labeling design is shown in Supplementary Table 1.

### 2.3. Quantitative Proteomic Analysis

TMT-labeled peptides (Thermo Fisher Scientific) were fractionated using the HPRP method and desalted before LC-MS/MS analyses. Technical details regarding instrument parameters and operational processes can be found in Supplementary Materials and Methods. The RAW files acquired were loaded into MaxQuant (version 1.6.1.0) and searched against the human UniProtKB database (88,473 sequences, version 09-2015). Andromeda was used as search engine for the identification of proteins. The database search was performed using the MS2 reporter ion mode with the 10plex TMT option. A mass tolerance of 7 ppm was set for the main database search. Trypsin with up to two missed cleavages was set. Oxidation (M) and carbamidomethyl (C) were set as variable and fixed modifications, respectively. An automatic decoy database search was performed. A protein level false-discovery rate (FDR) of 1% was set to filter the results.

For quantitative analysis, the TMT reporter ion intensity of each protein was first normalized against the median intensity of all proteins within each sample to correct label-to-label variations. Subsequently, it was normalized against the averaged reference ion intensities of 131N and 131C labels within each run to correct run-to-run variations. At least two unique peptides were required for protein quantitation. Proteins with empty values were discarded. Student's* t*-test was performed for each protein between groups with the Perseus software. Proteins that showed more than twofold change (fold change of >2 or <0.5) with P value < 0.05 were considered to show significant differential expression. When analyzing proteins involved in nicotinamide adenine dinucleotide (NAD) metabolism and signaling pathways, proteins that showed more than 1.2-fold change (fold change of >1.2 or <0.84) were considered to show significant change. Multivariate principal component analysis (PCA) and heat maps were used to summarize and visualize sample classification on the basis of expression profiles of all proteins.

### 2.4. Bioinformatics Analysis

ProteinAtlas was used to analyze the tissue specificity of proteins differentially expressed between patients with IBD and the controls. Differentially expressed proteins (DEPs) were then divided into 2 groups: gastrointestinal (GI) tissue-specific group (GI tissue types) and GI tissue-nonspecific group (other tissue types). Subsequently, reactome pathway analysis (https://www.reactome.org/) was used to cluster the pathways in which the 2 groups were involved. The pathway analysis results were visualized using a bubble chart. CD38 gene expression levels of patients with UC (datasets GDS3119 and GDS2642) and of mouse colitis models (datasets GDS4363 and GDS3859) were downloaded from the NCBI GEO database. The expression data were analyzed with the GraphPad Prism 6 software.

### 2.5. Mouse Model of Dextran Sulfate Sodium Salt-Induced Colitis

12 male C57BL/6J mice aged 6–8 weeks were purchased from the Zhejiang Academy of Medical Science. The mice were orally administered 4% dextran sulfate sodium salt (DSS; molecular weight: 36,000–50,000; MP Biomedicals, Santa Ana, CA, USA) in water for 5 days to induce acute colitis (n = 6/group). Control mice (n = 6/group) were given drinking water. Body weight and stool consistency were recorded every day. On the sixth day, the mice were euthanized with 5% chloral hydrate. The colons were resected and fixed immediately in 10% formalin and embedded. This study was performed according to the guidelines of the animal ethics committee of the First Affiliated Hospital of Zhejiang University School of Medicine.

### 2.6. Immunohistochemical Staining

Immunohistochemical (IHC) staining was performed on paraffin-embedded sections of patients who had undergone colon resection. Tissues were cut into 5 *μ*m-thick sections and stained with H&E before IHC staining. Slides were incubated with primary antibodies against ITLN1 (ab118232, Abcam) and CD38 (ab108403, Abcam) for humans and CD38 (sc374650, Santa Cruz) for mice overnight at 4°C, followed by incubation with horseradish peroxidase-conjugated anti-IgG for 1 h at 37°C. The samples were visualized with 3,3′-diaminobenzidine (DAB) and observed under a light microscope (Leica, Germany).

### 2.7. Immunofluorescence Staining

Paraffin-embedded sections of colon tissues from patients with IBD or controls were used. The slides were incubated with primary antibodies against CD38 (sc374650, Santa Cruz) and F4/80 (ab16911, Abcam) at 4°C overnight. On the next day, the slides were incubated with sheep anti-mouse Alexa Fluor 488 (DaWen Biotech, China) and goat anti-rat Cy3 (DaWen Biotech, China) antibodies at 37°C for 1 h, followed by incubation with 4′,6-diamidino-2-phenylindole (DAPI) for 3 min. Images were obtained using a laser scanning microscope (Olympus, Japan).

### 2.8. Western Blot Analysis

Specimens were homogenized in RIPA buffer and centrifuged at 12,000 rpm for 15 min at 4°C; subsequently, the supernatants were collected. Proteins (40 *μ*g) were separated using 10% sodium dodecyl sulfate (SDS)-polyacrylamide gel electrophoresis (PAGE) gels and were then transblotted onto 0.45 *μ*m nitrocellulose membranes (Millipore, Merck, Germany). The membranes were blocked with 5% nonfat milk. Primary antibodies against CD38 (ab108403, Abcam) and glyceraldehyde 3-phosphate dehydrogenase (GAPDH; 5174; Cell Signaling Technology) were used. After incubation with the secondary antibody, the bands were visualized using an electrochemiluminescence (ECL) imaging system.

### 2.9. Statistical Analysis

Experimental data have been expressed in terms of mean ± standard error of the mean (SEM) values, and the GraphPad Prism 6 software was used for comparison between groups. Unpaired Student's* t*-test was used to compare differences between two groups. For more than 2 groups, analysis of variance (ANOVA) was used. A two-sided P value <0.05 was considered to be statistically significant.

## 3. Results

### 3.1. Proteins Differentially Expressed between Controls and Patients with IBD

**Figure 1:**
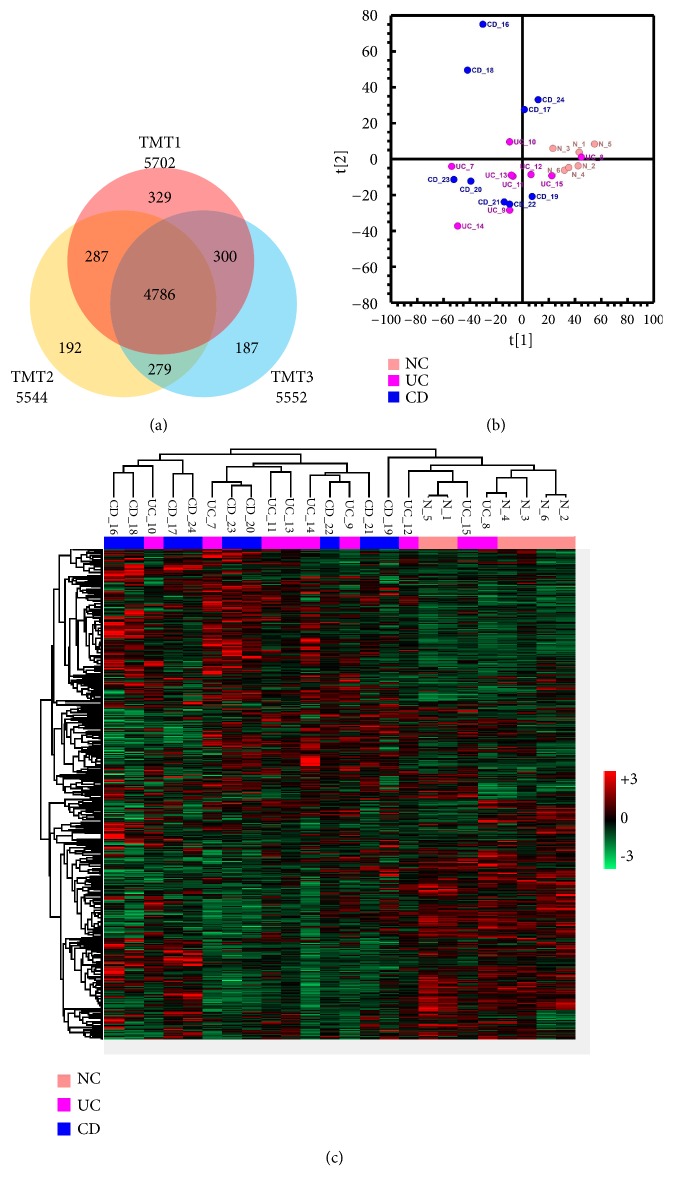
Summary of proteomics analysis of NC, CD, and UC using TMT quantitation method. (a) Venn diagram showing proteins identified across 3 TMT experiments, from which 4786 commonly identified proteins were used for downstream analyses. (b) Overall differences of proteome between NC, CD, and UC were summarized by PCA plot. (c) Heat map representation of abundance profiles of all 4786 proteins in all samples. Color shade correlates with relative protein abundances across each row (red/green for upregulation/downregulation).

**Figure 2:**
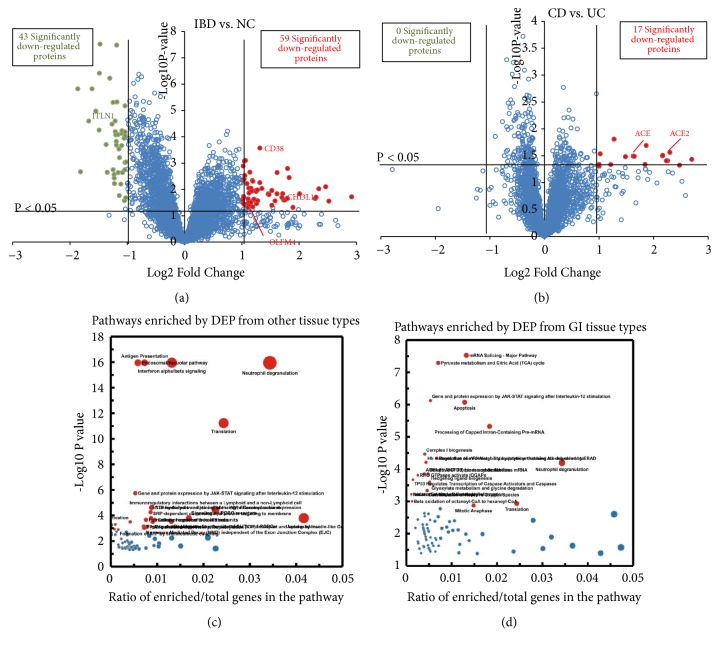
Identification and pathway analysis of DEPs. (a) Volcano map of DEPs between IBD and NC. (b) Volcano map of DEPs between CD and UC. (c) Reactome pathway analysis of DEPs enriched from no tissue types. (d) Reactome pathway analysis of DEPs enriched from GI tissue types.

We enrolled 6 controls, 9 patients with CD, and 9 patients with UC. Using a protein level FDR of 1% as a criterion, 5702, 5544, and 5552 proteins were identified from 3 TMT experiments (Figure 1(a)). A total of 4786 proteins were identified from the 3 experiments; of these, 102 were differentially expressed between patients with IBD and controls. The proteome between patients with IBD and controls could be clearly separated, as visualized by the PCA plot (Figure 1(b)) and heat map (Figure 1(c)). Among the DEPs, 59 proteins showed higher expression and 43 showed lower expression in patients with IBD than in controls (Figure 2(a)). Seventeen proteins were differentially expressed between patients with CD and patients with UC (Figure 2(b)). To analyze the related functions of proteins differentially expressed between patients with IBD and controls, reactome clustering pathway analysis was performed. Pathways enriched by DEPs from other tissues were mainly involved in the immune system, including the adaptive immune system and processes such as antigen presentation, interferon alpha/beta signaling, and the neutrophil degranulation pathway (Figure 2(c)). Pathways enriched by DEPs from GI tissue types included the mRNA splicing-major pathway, pyruvate metabolism and citric acid (TCA) cycle, gene and protein expression by JAK-STAT signaling after interleukin-12 stimulation, and apoptosis pathway (Figure 2(d)).

### 3.2. DEPs according to Disease Subtypes of Inflammatory Bowel Disease

102 proteins were significantly dysregulated in patients with IBD (Figure 2(a)). Of these, several proteins have been used as disease markers. Among them, lipocalin 2 (LCN2), S100A12, and matrix metallopeptidase 9 (MMP9) were upregulated in both UC and CD, while S100A8, S100A9, myeloperoxidase (MPO), and lactotransferrin (LTF) were upregulated in CD (Supplementary Table 2). Several novel proteins were also identified (Table 1). Chitinase 3-like 1 (CHI3L1), CD38 molecule (CD38), and olfactomedin 4 (OLFM4) were upregulated in patients with CD or UC, whereas intelectin 1 (ITLN1) was downregulated. We also identified proteins that were differentially expressed between the two subtypes of IBD. Our results indicated that 17 proteins were upregulated in CD compared to UC (Figure 2(b)). Some of the proteins with abundance changes are shown in Table 2; from these, angiotensin converting enzyme 2 (ACE2) and angiotensin converting enzyme 1 (ACE) showed significantly higher expression in CD than in UC.

### 3.3. NAD Metabolism and Signaling Pathway Showed Alterations in Patients with IBD

**Figure 3:**
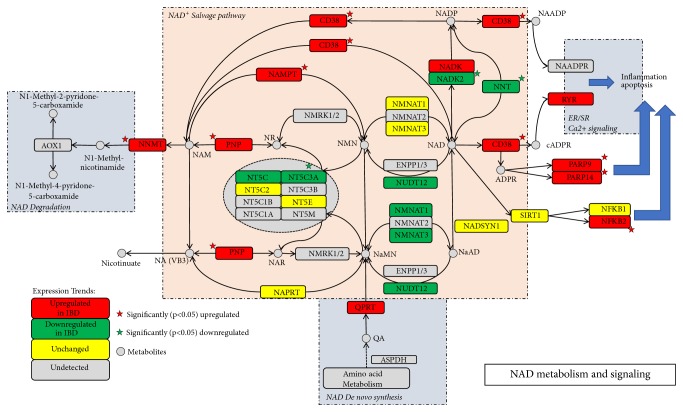
The role and expression of proteins identified by proteomic analysis within NAD+ metabolism and signaling pathway. Red (green) rectangle represents proteins upregulated (downregulated) in IBD. Red (green) rectangle with red pentagram represents proteins upregulated (downregulated) in IBD with p value < 0.05. Yellow rectangle represents proteins that are unchanged between IBD and controls. Gray rectangle represents proteins undetected by proteomic analysis.

Using reactome pathway analysis, we noted that many proteins are involved in immune pathways. Previous studies have revealed that CD38 plays multiple functions in rheumatoid arthritis (RA), allergic airway disease, and multiple myeloma and is expressed in the membrane of immune cells [21–23]. Furthermore, Michael reported that CD38 is expressed on inflammatory cells of the intestine and promotes intestinal inflammation [24]. Therefore, we studied the role of CD38 in IBD. CD38 participates in the synthesis of cyclic ADP ribose (cADPR) from NAD, and the NAD metabolism pathway is reported to promote inflammation in the gut [24, 25]; therefore, we analyzed our proteomic results for the expression of other molecules involved in NAD^+^ metabolism and signaling. Twenty-two proteins involved in NAD metabolism and signaling were identified by the proteomic analysis (Table 3). When the fold change level was set at >1.2 or <0.84; 10 of them showed significant change (Table 3); most of these were enzymes related to NAD synthesis and cleavage. The role and expression of these proteins within the NAD metabolism and signaling pathway are indicated in Figure 3.

### 3.4. CD38 Expression Increased in Patients with IBD and in the Mouse Colitis Models

**Figure 4:**
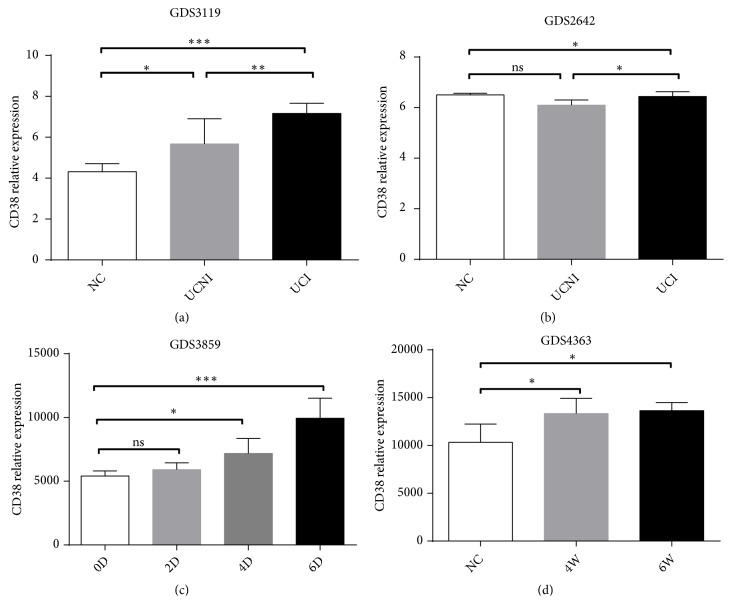
CD38 mRNA expression in patients with UC and animal colitis models from GEO datasets. (a) Colon mucosa CD38 mRNA expression in NC, UCI, and UCNI patients based on GEO GDS3319 dataset. (b) Colon mucosa CD38 mRNA expression in UCI and UCNI patients based on GEO GDS2642 dataset. (c) Colon CD38 mRNA expression in DSS induced mouse colitis model at 0D, 2D, 4D, and 6D based on GEO GDS3859 dataset. (d) Colon CD38 mRNA expression in T cell transfer mouse colitis model at 0W, 4W, and 8W based on GEO GDS4363 dataset. Significance level: *∗*P < 0.05, *∗∗*P < 0.01, and *∗∗∗*P < 0.001. NC, normal control; CD, Crohn's disease; UC, ulcerative colitis; UCI, ulcerative colitis inflamed; UCNI, ulcerative colitis noninflamed; D, day; W, week.

We analyzed CD38 mRNA expression by using the NCBI GEO database. The expression data in the GDS3119 database indicated that the inflamed tissues in UC had higher CD38 expression than the controls (Figure 4(a)). The CD38 expression in the inflamed regions was higher than that in the noninflamed regions (Figures 4(a) and 4(b)). In the DSS-induced colitis model and T cell transport colitis model, CD38 expression gradually increased with the emergence of colitis (Figures 4(c) and 4(d)).

**Figure 5:**
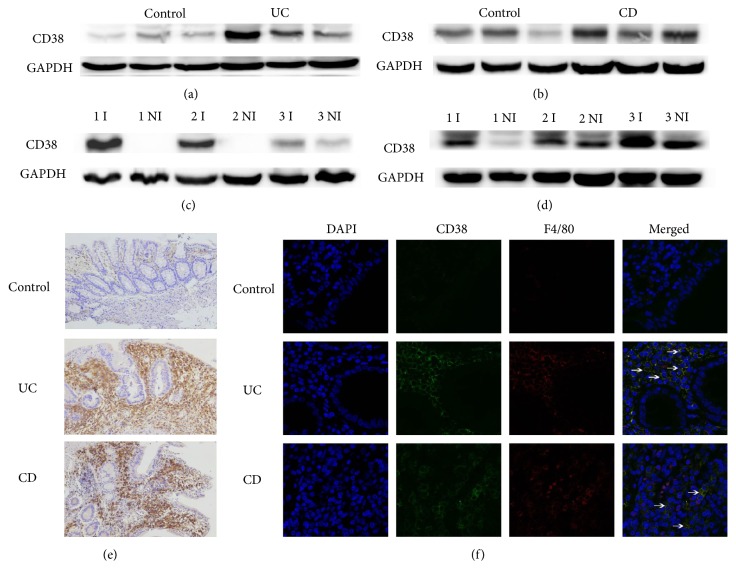
CD38 is increased in IBD patients. (a) Western blot analysis of CD38 expression in control and UC patients (N=3 per group). (b) Western blot analysis of CD38 expression in 3 matched pairs of inflamed (I) and noninflamed (NI) UC tissues. (c) Western blot analysis of CD38 expression in control and CD patients (N=3 per group). (d) Western blot analysis of CD38 expression in 3 matched pairs of inflamed (I) and noninflamed (NI) CD tissues. (e) Expression of CD38 by IHC (original magnification×100). (f) Immunofluorescence staining of DAPI (blue), CD38 (green), and F4/80 (red) in control, UC, and CD patients (N=3 per group) (original magnification, ×200). CD, Crohn's disease; UC, ulcerative colitis.

We then analyzed CD38 protein expression in colon specimens from patients with CD or UC. CD38 protein expression was higher in patients with CD or UC than in controls (Figures 5(a) and 5(b)). The CD38 expression in inflamed regions was higher than that in noninflamed regions (Figures 5(c) and 5(d)). IHC and immunofluorescence (IF) staining confirmed increased CD38 expression and membrane CD38 distribution (Figures 5(e) and 5(f)). We noted colocalization of CD38 with the macrophage marker F4/80 (Figure 5(f)). CD38 protein expression also increased in the mice with DSS-induced colitis (Supplementary Figure 1).

## 4. Discussion

Since the first study by Barceló-Batllori et al. [26], who identified increased indoleamine-2,3-dioxygenase expression in cytokine-treated colon epithelial cells by using proteomics technology, numerous studies have investigated proteomic changes in IBD. We previously identified several protein peaks in relation to serum samples, which were helpful for differentiating CD from intestinal tuberculosis (ITB) [27]. Isobaric chemical labeling for quantitative proteomics has better quantification performance and reproducibility than other proteomic methods [28]. In this study, we employed TMT-based quantitative proteomics to identify DEPs in patients with IBD.

We identified several previously reported proteins, such as S100A8/9, S100A12, LTF, LCN2, and MMP9, most of which are used to evaluate the disease activity of IBD [29–32]. We also identified several novel proteins associated with IBD; CH3L1, CD38, and OLFM4 showed increased levels, whereas ITLN1 showed decreased levels. Previous DNA microarray analysis has shown that CH3L1 is upregulated in inflamed mucosa [33]; our result is consistent with these microarray results. Several studies also revealed that fecal CHI3L1 aids in predicting the severity and activity of intestinal inflammation in both pediatric and adult IBD [34, 35]. However, fecal CHI3L1 analysis has not been analyzed in Asian populations. OLFM4 protein expression was found to increase by 1.7 folds in CD and 3.7 folds in UC [36], which is similar to our results. ITLN1 is a lactoferrin receptor that can recognize microbial glycans in the intestine [37]. Previous studies revealed that serum ITLN1 levels decrease in IBD and are negatively correlated with its disease activity [38]. However, the role of ITLN1 in IBD pathogenesis is still unclear.

Differential diagnosis between CD and UC is important for guiding treatment and follow-up. In the current study, we identified 17 proteins that showed differences in expression between CD and UC. Among these, ACE2 and ACE showed much higher expression in patients with CD than in patients with UC. Both ACE2 and ACE are associated with the development of organ fibrosis [39, 40] and CD is characterized by subepithelial fibrosis in some patients, which might explain the increased ACE2 and ACE levels in CD. However, the functions of the proteins identified were unclear and need to be confirmed in future studies.

Proteomic analysis indicated that the expression of many proteins involved in NAD metabolism and signaling showed changes, suggesting that NAD metabolism and signaling are associated with the gut inflammation noted in IBD. NAD is a major coenzyme in bioenergetic processes, including oxidative phosphorylation and energy homeostasis [41]. NAD is also the substrate for NAD-cleaving enzymes such as poly (ADP-ribose) polymerases (PARPs), sirtuins (SIRTs), and cADP-ribose synthases such as CD38 [42–44]. NAD cleavage by these enzymes is important for many physiological processes. NAD synthesis consists of two pathways, the de novo synthesis pathway and salvage synthesis pathway, with the latter playing an important role in mammals. In the salvage pathway, NAMPT is the key enzyme catalyzing NAD synthesis. A previous proteomic study revealed that NAMPT levels increase in the inflamed colonic mucosa of patients with IBD [45], which was also confirmed by our study. Romana et al. reported that the NAMPT inhibitor FK866 alleviates the PARP/SIRT-mediated inflammatory response and alters macrophage polarization in DSS-induced colitis in mice [25]. NAMPT inhibition leads to decreased CD38^+^ immune cell infiltration into the inflamed colon. However, the roles of the other enzymes identified by our study in the pathogenesis of colitis are not clear.

The CD38 expression level has been further confirmed by validation studies. CD38 is an ectoenzyme that catalyzes the synthesis of cADPR and NAADP from NAD+ [46]. CD38-cADPR signaling can mediate airway hyperresponsiveness by increasing calcium release in airway smooth muscle cells [22]. CD38 is also involved in multiple myeloma; antibodies against CD38, including daratumumab and MOR202, are promising therapeutics for multiple myeloma [23]. Using microarray analysis, Chang et al. [21] found that CD38 increased in RA synovial tissues. Recently, a study using RNA sequencing also revealed that CD38 was significantly upregulated in the synovial tissue of patients with RA at various stages [47]. Furthermore, their ex vivo experiments showed that daratumumab effectively depletes plasma cells in peripheral blood mononuclear cells (PBMCs) and that CD38 inhibition can be a novel treatment option for both RA and systemic lupus erythematosus (SLE). CD38^−/−^ mice have shown decreased immune cell infiltration and mild colitis symptoms upon DSS treatment [24]. Shu et al. reported that CD38 expression increased in macrophages upon LPS stimulation and CD38 suppression inhibited macrophage M1 polarization and activation of nuclear factor-*κ*B (NF-*κ*B) signaling [48], suggesting that CD38 expression in macrophages is proinflammatory. Our results showed that CD38 was localized in F4/80^+^ macrophages; however, we could not exclude the distribution of CD38 within other cell types. The molecular mechanisms underlying the effect of CD38 in intestinal macrophages in colitis require further research.

## 5. Conclusion

Using TMT proteomic quantification, the current study identified proteins that were differentially expressed between patients with IBD and controls. We found that proteins involved in the NAD metabolism and signaling pathway showed significant alterations in IBD; of these, the expression of CD38 was validated. Further studies are required to clarify the mechanisms underlying the promotion of intestinal inflammation by CD38 and to determine whether CD38 inhibition can be used as a treatment option for IBD.

## Figures and Tables

**Table 1:** Novel proteins showing abundance changes in CD or UC patients.

UniProt accession	Protein	UC/Con		CD/Con		CD/UC		Unique peptides	GI related or not
		Fold change	p value	Fold change	p value	Fold change	p value		
P36222	CHI3L1	3.42	0.0434	3.13	0.0048	0.92	0.7712	4	not
P28907	CD38	2.28	0.0015	2.70	0.0004	1.18	0.2881	9	not
Q6UX06	OLFM4	2.32	0.0779	1.89	0.0030	0.81	0.4557	5	not
Q8WWA0	ITLN1	0.50	0.0048	0.36	0.0000	0.73	0.1718	6	yes

CD, Crohn's disease. UC, ulcerative colitis. Con, control. GI, gastrointestinal.

**Table 2:** Proteins showing abundance changes between CD and UC patients.

UniProt accession	Protein	UC/Con		CD/Con		CD/UC		Unique peptides	GI related or not
		Fold change	p value	Fold change	p value	Fold change	p value		
P05062	ALDOB	1.55	0.1156	8.62	0.0843	5.56	0.0477	17	not
Q9BYF1	ACE2	1.30	0.2869	6.41	0.0575	4.91	0.0274	2	not
P12104	FABP2	1.02	0.9457	4.87	0.0912	4.78	0.0394	9	not
P12821	ACE	1.26	0.0372	3.96	0.0580	3.13	0.0324	12	not

CD, Crohn's disease. UC, ulcerative colitis. Con, control. GI, gastrointestinal.

**Table 3:** Expression of proteins involved in NAD metabolism and signaling pathway within the proteomic results.

UniProt accession	Protein	p value	Fold change	Unique	GI related or not
			IBD/Con	peptides	not
P36222	CD38	0.0030	2.49	9	not
O95544	NADK	0.0768	1.43	5	not
Q4G0N4	NADK2	0.0183	0.82	6	not
Q6IA69	NADSYN1	0.7181	0.97	3	not
C9JF35	NAMPT	0.0084	1.77	14	not
Q6XQN6	NAPRT	0.3029	0.88	14	not
Q9HAN9	NMNAT1	0.112	0.87	3	not
Q96T66	NMNAT3	0.1096	0.79	2	not
P40261	NNMT	0.0013	1.73	6	not
Q13423	NNT	0.0023	0.79	4	yes
Q8TCD5	NT5C	0.1245	0.83	6	not
P49902	NT5C2	0.4623	0.94	4	not
Q9H0P0	NT5C3A	0.0288	0.67	7	not
P21589	NT5E	0.7597	0.92	3	not
Q9BQG2	NUDT12	0.0875	0.82	2	not
P00491	PNP	0.0131	1.34	10	not
Q15274	QPRT	0.1998	1.47	4	not
Q96EB6	SIRT1	0.1482	1.19	2	not
Q8IXQ6	PARP9	0.0038	1.53	9	not
Q460N5	PARP14	0.0013	1.52	12	yes
Q00653	NFKB2	0.0446	1.65	4	not
P19838	NFKB1	0.7749	1.02	11	not

IBD, inflammatory bowel disease. Con, control. GI, gastrointestinal.

## Data Availability

The data used to support the findings of this study are available from the corresponding author upon request.

## Supplementary Materials

Supplementary 1 Materials and Methods: technical details regarding the instrument parameters and operational process of TMT labeling, high pH reversed phase fractionation, and LC-MS/MS analysis.

Supplementary 2 Table 1: the TMT-labeling design for controls, patients with UC, and patients with CD.

Supplementary 3 Table 2: proteins used as disease markers and upregulated in UC or CD.

Supplementary 4 Figure 1: CD38 protein expression increased in mice with DSS-induced colitis.

Supplementary 5 Excel files: quantitation values, significance in the different comparisons performed, peptides identified, and so forth.
