# Supplementary material for: Quantitative Proteomic Analysis Reveals the Deregulation of Nicotinamide Adenine Dinucleotide Metabolism and CD38 in Inflammatory Bowel Disease
Source: Biomed Res Int. 2019 Apr 23;2019:3950628. doi: 10.1155/2019/3950628 (PMC6507272; doi:10.1155/2019/3950628)
Supplement: Supplementary 1 — Materials and Methods: technical details regarding the instrument parameters and operational process of TMT labeling, high pH reversed phase fractionation, and LC-MS/MS analysis. [file 3950628.f1.doc]

**Supplementary Materials and Methods**

**TMT labeling**

Digested peptides from each sample were mixed with TMT label dissolved in 41 ul anhydrous acetonitrile and were incubated for 2h at room temperature. The reaction was stopped by 100 μL of NH_4_HCO_3_(final concentration at 200mM). All samples were then pooled within each TMT experiment and concentrated in vacuum concentrator before HPLC fractionation.

**High PH reverse phase fractionation and LC-MS/MS analysis**

The TMT multiplexed peptides were separated on a 1260 HPLC System (Agilent) equipped with an Acquity Peptide BEH C18 column (1.7 μm, 130 Å, 2.1 mm × 150 mm, Waters). Mobile phase A contains 0.1% NH_4_OH and B contains 0.1% NH_4_OH in ACN. The 60 min solvent gradient at a flow rate of 0.2 mL/min was set as follows: 5% B within 2 min; 5−18% B in 35 min; 18−32% B in 15 min; 32−95% B in 3 min; maintained at 95% B for 5 min. Fractions were collected every 1 min. For each TMT experiment, 60 collected fractions were combined into 15 fractions via a concatenated fashion and then were dried by Speed Vac.

For each fraction, around 500 ng of peptides suspended in 0.1% FA in 2% ACN were enriched on a Symmetry C18 nanoACQUITY Trap Column (100 Å, 5 µm, 180 µm x 20 mm). Peptide separation was performed by a BEH C18 nanoACQUITY Column (130Å, 1.7 µm, 75 µm X 250 mm) on an nanoACQUITY UPLC system (Waters, Milford, MA) operated at 200 nL/min. The gradient started with 2% of ACN and increased to 5% ACN in 10 min, then reached 19% ACN in 70 min and 30% ACN in 15 min. The gradient finally reached 98% ACN in 10 min and was then held for 5 min before it returns to 2% ACN in 2 min and kept at the re-equilibration condition for 8 min. The total analysis time per injection was 120 min. Both mobile phases were all supplemented with 0.1% FA.

The nanoLC was coupled to an Orbitrap Q-Exactive mass spectrometer (Thermo). The nanospray source was operated at 2.0 kV. The MS was operated in data-dependent analysis (DDA) mode scheduling a full MS survey scan at the 70,000 FWHM resolution (at m/z 200 Th) with automatic gain control (AGC) set to 3e6, followed by 20 MS^2^ scans of precursors selected for fragmentation by higher-energy collision dissociation with normalized collision energy set to 28%. Dynamic exclusion was set to 40 sec. All MS^2^ spectra were acquired at 35,000 FWHM resolution with AGC of 2e5.
