# Supplementary material for: Quantitative Proteomic Analysis Reveals the Deregulation of Nicotinamide Adenine Dinucleotide Metabolism and CD38 in Inflammatory Bowel Disease
Source: Biomed Res Int. 2019 Apr 23;2019:3950628. doi: 10.1155/2019/3950628 (PMC6507272; doi:10.1155/2019/3950628)
Supplement: Supplementary 2 — Table 1: the TMT-labeling design for controls, patients with UC, and patients with CD. [file 3950628.f2.docx]

**Supplementary Table 1 TMT10plex labeling design**

| Isobaric labeling | Run1 | Run2 | Run3 |
| --- | --- | --- | --- |
| TMT-126 | NC4 | NC5 | NC6 |
| TMT-127N | NC1 | NC2 | NC3 |
| TMT-127C | UC6 | UC8 | UC9 |
| TMT-128N | UC4 | UC5 | UC6 |
| TMT-128C | UC1 | UC2 | UC3 |
| TMT-129N | CD6 | CD8 | CD9 |
| TMT-129C | CD4 | CD5 | CD6 |
| TMT-130N | CD1 | CD2 | CD3 |
| TMT-130C | reference | reference | reference |
| TMT-131 | reference | reference | reference |
