# Supplementary material for: Quantitative Proteomic Analysis Reveals the Deregulation of Nicotinamide Adenine Dinucleotide Metabolism and CD38 in Inflammatory Bowel Disease
Source: Biomed Res Int. 2019 Apr 23;2019:3950628. doi: 10.1155/2019/3950628 (PMC6507272; doi:10.1155/2019/3950628)
Supplement: Supplementary 3 — Table 2: proteins used as disease markers and upregulated in UC or CD. [file 3950628.f3.docx]

**Supplementary Table 2 Proteins used as disease markers and upregulated in UC or CD**

| Uniprot accession | Protein | UC/Con  Fold change p value | | CD/Con  Fold change p value | Unique  peptides | GI related or nott |  |
| --- | --- | --- | --- | --- | --- | --- | --- |
| P80188  P14780  P05109  P06702  P80511  P02788  P05164 | LCN2  MMP9  S100A8  S100A9  S100A12  LTF  MPO | | 3.71 0.0136  2.71 0.0355  4.23 0.0594  2.61 0.1147  3.25 0.0193  3.51 0.1063  3.05 0.0574 | 4.33 0.0280  3.93 0.0238  5.59 0.0123  3.36 0.0067  3.70 0.0002  3.92 0.0312  3.88 0.0166 | 7  11  10  7  12  25  15 | not  not  not  not  not  not  not | |

CD, Crohn’s disease; UC, ulcerative colitis. Con, control. GI, gastrointestinal.
