# Supplementary material for: Quantitative Proteomic Analysis Reveals the Deregulation of Nicotinamide Adenine Dinucleotide Metabolism and CD38 in Inflammatory Bowel Disease
Source: Biomed Res Int. 2019 Apr 23;2019:3950628. doi: 10.1155/2019/3950628 (PMC6507272; doi:10.1155/2019/3950628)
Supplement: Supplementary 4 — Figure 1: CD38 protein expression increased in mice with DSS-induced colitis. [file 3950628.f4.docx]

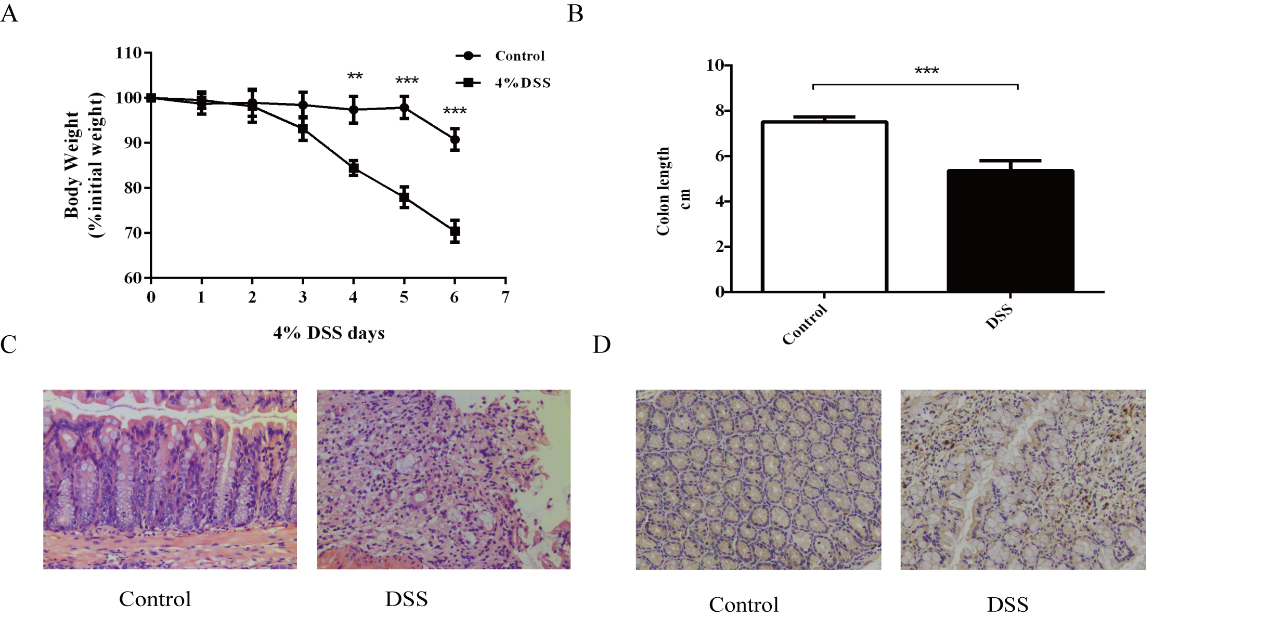


**Supplementary Figure 1** CD38 is increased in DSS induced mouse colitis. (A) Body weight changes in control group and DSS group. (2) Colon length between control and DSS groups. (C) HE staining of colon tissues of control and DSS mice. (D）Expression of CD38 in control and DSS mice by IHC. N=6 in each group. Data were expressed as mean±SEM. *P < 0.05, **P < 0.01, ***P < 0.01.
